# Relationship between X-ray findings of lumbar spondylosis and knee pain

**DOI:** 10.1186/s12891-019-2755-1

**Published:** 2019-08-17

**Authors:** Kosuke Uehara, Masami Akai, Tokuhide Doi, Hiroyuki Oka, Tsutomu Iwaya

**Affiliations:** 1 Rehabilitation Hospital, National Rehabilitation Center, Saitama, Japan; 2 Departments of Orthopaedic Surgery, University of Tokyo Hospital, 7-3-1, Hongo, Bunkyo-ku, Tokyo, Japan; 3 Graduate school, International University of Health and Welfare, Tokyo, Japan; 4 Geriatric Care Facility Narita-tomisato Tokushuen, Tomisato, Japan; 5 Department of Medical Research and Management for Musculoskeletal Pain, 22nd Century Medical & Research Center, The University of Tokyo, Tokyo, Japan

**Keywords:** Knee pain, Disc height, Knee-spine syndrome, Lumbar spondylosis, Locomotive syndrome, Lumbar spine, Disc degeneration, Elderly people

## Abstract

**Background:**

The aim of this study was to investigate the correlation between radiographic measurement in lumbar spine and clinical information including symptoms or results of functional testing using a baseline data of longitudinal cohort study.

**Methods:**

A total of 314 elderly subjects were recruited from 5 orthopedic clinics or affiliated facilities. Data for the present investigation were collected via an interviewer-administered questionnaire, which included questions on past medical history, drug history, pain area. And also results of functional testing and X-ray imaging of the lumbar spine were collected. Analysis was carried out to determine any correlation between results of X-ray imaging of the lumbar spine and other collected data, and sorted regarding Akaike Information Criterion (AIC). The correlations among these variables and odds ratio were also analyzed.

**Results:**

T12/L1% disc height showed a minimum AIC value with buttock pain (− 4.57) and history of vertebral fracture (− 4.05). The L1/L2, L2/L3, and L3/L4% disc height had a minimal AIC value with knee pain (− 4.11, − 13.3, − 3.15, respectively), and odds ratio of knee pain were 3.5, 3.8, and 2.7, respectively.

**Conclusions:**

Correlation was recognized between the T12/L1% disc height and both buttock pain and previous vertebral fractures, and the L1/L2, L2/L3, and L3/L4% disc height showed a correlation with knee pain. Especially the L2/L3% disc height and knee pain had a strong correlation. It was suggested that these findings may provide additional basis to the concept that lumbar spinal lesion associates with knee pain clinically.

**Electronic supplementary material:**

The online version of this article (10.1186/s12891-019-2755-1) contains supplementary material, which is available to authorized users.

## Background

Musculoskeletal conditions, such as lumbar spondylosis is major public health issues and important causes of physical impairment among the elderly populations of most developed countries [1]. To evaluate lumbar spondylosis, plain radiograph is considered the gold standard as a method that is less-invasive, inexpensive, and convenient. On lumbar radiographic features, disc height is strongly correlated with disc degeneration classified from MR images [2]. Furthermore, previous studies revealed that each lumbar disc level had differences in the symptoms and clinical course of patients [3, 4]. Thus, to examine lumbar radiographic factors associated with symptoms, each disc height should be assessed separately. To understand the essential characteristics of such multifaceted entities, statistical and mathematical tools are available to define the assessing conditions. The aim of this study was to investigate the correlation between radiographic measurements in lumbar spine and clinical information.

## Methods

### Participants

All participants provided written informed consent, and the study was conducted with the approval of the appropriate ethical committees. This research was in compliance with the Helsinki Declaration.

The Locomotive Disability Prevention (LDP) study is a prospective study to establish epidemiologic indexes for evaluation of clinical evidence for the development of rehabilitation programs at 5 orthopedic clinics or affiliated nursing care facilities. We recruited a total of 314 elderly subjects, whose ages ranged from 65 to 93 (mean 77.4 years old), each of whom joined outpatient rehabilitation programs at one of the above facilities.

Inclusion criteria were as followings: 1. Age ≥ 65 years, 2. Any 1 of the following 4 criteria; Complaints related to the legs or spine without disability in walking or leaving the home (outpatients); Complaints related to the legs and spine, and slight disabilities in walking and leaving the home (outpatients); Slight disability in walking due to locomotive organ disorders (users of long-term care services); Complaints related to the upper extremities without disability in walking or leaving the home (outpatients at orthopedic clinics), 3. Ability to answer the Geriatric Locomotive Function Scale-25 (GLFS-25) questionnaire without assistance, and 4. Consent to radiographic examination, blood test, and function test.

Exclusion criteria were as followings: 1. Inability to stand up from a chair or bed, 2. Disability in walking or locomotion because of neurological disease requiring admission treatment. 3. Severe pulmonary, renal, coronary, or hepatic disease, 4. Mental illness, 5. Past history of stroke within the preceding 6 months, 6. Past history of myocardial infarction within the preceding 6 months, 7. Past history of fracture of a lower extremity within the preceding 6 months, 8. Current treatment for acute trauma, and 9. Other reasons determined by the attending physician.

### Data collection

The LDP study started on April, 2009 for base line data and finished on March, 2011 for collecting the 24 month data. After the baseline data were obtained, the data of 6, 12, 18, and 24 month were collected. For this study, we analyzed only baseline data.

Participants completed an interviewer-administered questionnaire consisting of 18 items, which included questions on living environment, past medical history, drug history, and pain area (back pain, buttock pain, thigh pain, knee pain) (Additional file 1). The questionnaire interviews were conducted by five orthopaedic surgeons, each with about 20- to 30- years’ experience. All of the participants had previous association with some of the doctors as patients. The GLFS-25 questionnaire was also answered as a patient oriented comprehensive assessment [5]. Information regarding pain areas was taken by interview by well-experienced orthopedic doctors, who asked, “In the past month, have you experienced knee pain?” and the same for lumbar spine, buttock, and thigh area respectively. The questionnaire interview was conducted at all time points at clinics, and they were not audio-recorded. The questions asked were all closed. Therefore, we performed only a descriptive statistical analysis of closed responses. These data and findings were synthesized with other data using allocated identification numbers only for this study.

To measure physical performance, one leg standing time, grip strength, one hundred stamping time test, and leg extension power were collected. The one leg standing time with eyes open measured the time for which the subject could remain standing on one leg, and was measured for both sides. The mean of both sides was calculated. The grip strength was measured for both sides using a Smedley style dynamometer, and the value of the stronger side was designated as the grip strength. The one hundred stamping time test measured the time needed to step in place 100 times, and the shorter value of 2 trials was designated as the one hundred stamping test time. The leg extension power was measured for both sides using a previously described device [6], and the mean value of both sides was designated as the leg extension power.

### Radiographic assessment

**Fig. 1:**
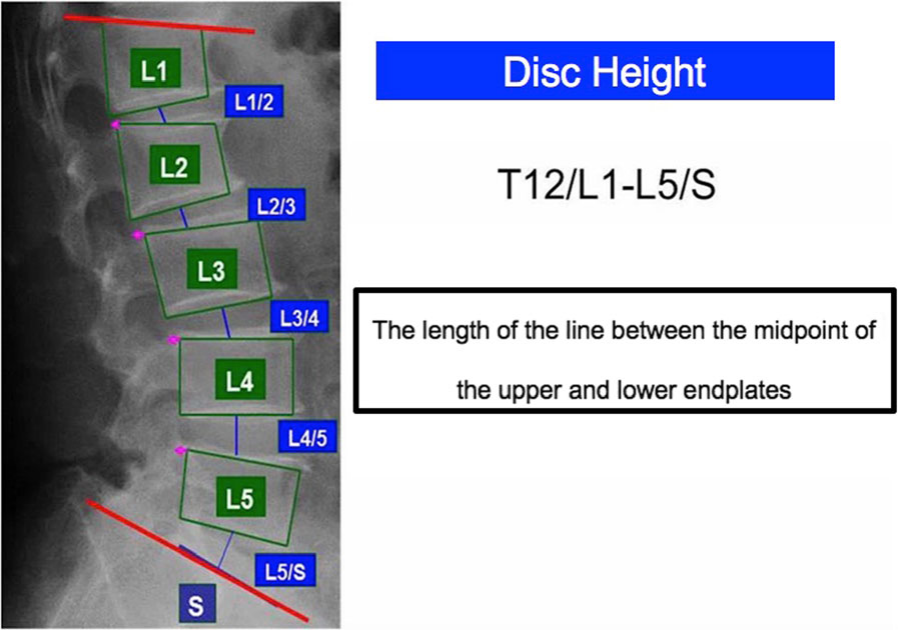
The Lumbar-Spine Computer-Aided Diagnostic system draws the vertebra in outline and measures the disc height of anterior, midpoint, and posterior semi-automatically. As for disc height in this study, the length of the line between the midpoint of the upper and lower endplates divided by the L3 posterior vertebral height was used

All participants underwent radiographic examination of the anteroposterior and lateral views of the lumbar spine, including intervertebral levels T12/L1 to L5/S, was performed. Using lateral view of the standing lumbar spine, Lumbar-Spine Computer-Aided Diagnostic system provided the disc height of the T12/L1-L5/S1, posterior wall height of each vertebra semi-automatically, and the results were confirmed that all the results were obtained correctly by H.O (Fig. 1). Disc height might be influenced by each vertebral size, so we standardized the disc height of the T12-S divided by the posterior wall height of the L3 vertebra, which is usually preserved in the original shape [7]. As a result, we could get a standardized % disc height for each intervertebra.

$$ \%\kern0.5em \mathrm{disc}\ \mathrm{height}=\left(\mathrm{disc}\kern0.5em \mathrm{height}/\mathrm{L}3\kern0.5em \mathrm{posterior}\kern0.5em \mathrm{wall}\kern0.5em \mathrm{height}\right)\times 100 $$

The Lumbar-Spine Computer-Aided Diagnostic system divided the vertebra shape into 4 groups: normal, wedge, compound, and fish; and the results were confirmed by H.O. The wedge, compound, and fish shapes were regarded as vertebral body fractures. Wedge was defined as when anterior vertebral height divided by posterior vertebral height was much less than 0.75. Compound was defined as when the anterior vertebral height of one vertebra divided by the anterior vertebral height of the one upper vertebra, and the posterior vertebral height of the first vertebra divided by posterior vertebral height of the one upper vertebra were both much less than 0.80.

### Data mining and statistical analysis

Statistical tools are available to understand the essential characteristics of multifaceted entities. Akaike Information Criterion (AIC) is a useful and excellent tool for analyzing latent structures within outcome measures [8]. We used the AIC to select the best model from among each % disc height (six discs: T12/L1 to L5/S) for objective variables. The following variables were included as explanatory variables; living environment, past medical history, drug history, and pain area (back pain, buttock pain, thigh pain, knee pain), GLFS-25, one leg standing time, grip strength, one hundred stamping time test, and muscular power of lower limb extension. The smaller the AIC value for a model, the more likely it is that the model fits the data best. The AIC value represents the amount of association between two items without latent confounding effects [9], and a negative AIC value indicates a stronger association between two items [8]. Using a special program with R language, some continuous variables were converted into categorical variables [10]. The results of analyses were shown for optimal sorting with CATDAP2 (CATegorical Data Analysis Program 2) in all the double and triple combinations among examined data as a round robin to investigate their relationships [9]. Values of *P* < 0.05 were considered to indicate statistical significance. As a next step, correlations among these variables, cut-off point of each % disc height, and odds ratio were also analyzed. Cut-off values of % disc height for the item that has correlation with knee pain were analyzed using AIC [5]. We made variables that divided into two categories by all of the sequential number of one decimal point order from minimal to maximum value of the each % disc height, and using CATDAP2 program that mentioned above, we analyzed the cut-off point that fit the best. Odds ratio were calculated with these cut-off values. Data analyses were performed using SPSS version 18 (SPSS Inc., Chicago, IL, USA).

## Results

**Fig. 2:**
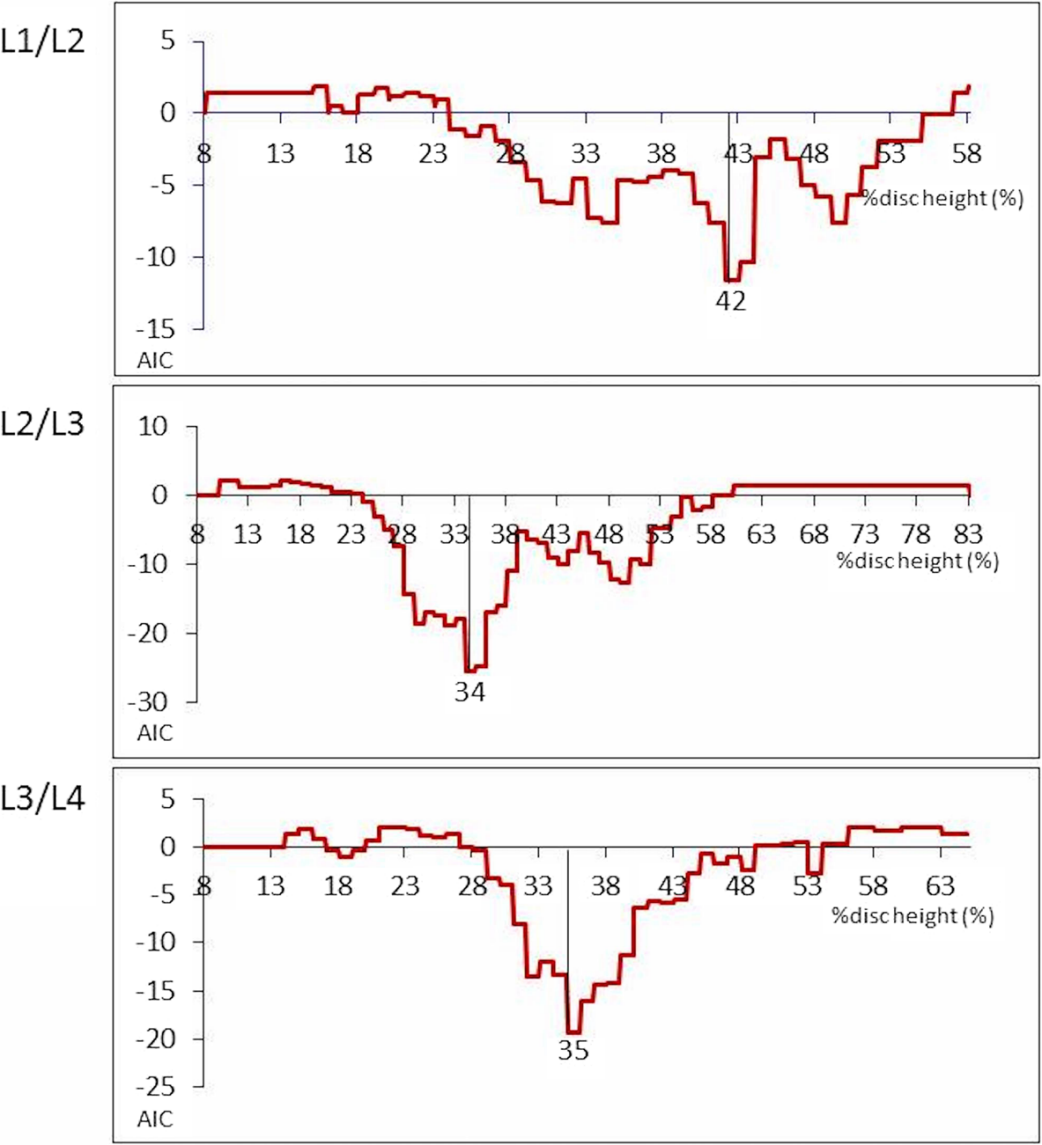
Cut-off values were shown as minimal AIC indicated when the presence or absence of knee pain was an objective variable. The lowest AIC value represents the model with the best fit

Table 1 shows selected characteristics of the participants including age, gender, pain area, functional tests, GLFS-25, and each % disc height. The results of CATDAP2 program using AIC showed the L1/L2% disc height, L2/L3% disc height, and L3/L4% disc height were strongly correlated with knee pain. Among these three disc heights, L2/L3% disc height had the strongest correlation, as AIC was − 13.3 (Table 2). The T12/L1% disc height showed a relation to buttock pain and previous vertebral fractures. L1/L2% disc height, L2/L3% disc height, and L3/L4% disc height also showed significant correlation with knee pain by Mann-Whitney test (*P* < 0.001). Cut-off values were shown as minimal AIC indicated when the presence or absence of knee pain was an objective variable. Cut-off values were 42% in L1/L2% disc height, 34% in L2/L3% disc height, and 35% in L3/L4% disc height, respectively (Fig. 2). Odds ratio of knee pain in the above cases were 3.5 (95% confidence interval (CI): 1.7–7.3), 3.8 (95% CI: 2.6–6.3), and 2.7 (95% CI: 1.6–4.5), respectively.

**Table 1:** Characteristics of the Study Participants

Measure	*n* = 314	
Age (yr)	77.4	± 6.4
Gender (females)	234	(74.5%)
Back pain	138	(43.9%)
Buttock pain	94	(29.9%)
Thigh pain	44	(14.0%)
Knee pain	170	(54.1%)
GLFS-25^†^	48.0	± 15.8
One leg standing time (s)	18.5	± 19.8
Grip strength (kg)	21.1	± 6.87
One hundred stamping time test (s)	58.2	± 11.9
Muscular power of lower limb extension (kg)	53.1	± 29.6
T12/L1% disc height* (%)	27.5	± 7.9
L1/L2% disc height* (%)	33.4	± 18.2
L2/L3% disc height* (%)	35.6	± 21.9
L3/L4% disc height* (%)	36.4	± 9.9
L4/L5% disc height* (%)	34.7	± 11.3
L5/S % disc height* (%)	29.1	± 10.2

The data are presented as the mean ± standard deviation or n (%). ^†^GLFS-25 = Geriatric Locomotive Function Scale-25; *% disc height = (disc height / L3 posterior wall height) × 100

**Table 2:** The results of AIC values with each % disc height

disc	Item 1	Item 2	AIC*
T12/L1	Buttock pain		−4.57
	Previous vertebral fracture		−4.05
	Previous vertebral fracture	Buttock pain	−1.58
	Lower limb pain		−1.11
L1/L2	Knee pain		−4.11
	Knee pain	Abnormal Posture	−1.31
L2/L3	Knee pain		−13.3
	Knee pain	Back pain	−6.14
L3/L4	Knee pain		−3.15
L4/L5	None		-- < 0
L5/S	None		-- < 0

*AIC = Akaike Information Criterion; Valuable are sorted in ascending order. The lowest AIC value represents the model with the best fit

## Discussion

For better understanding of orthopedic problems in elderly people, we conducted the present study and tried to investigate the relationship among pain characteristics, functional testings, physical activities, various parameter from radiographic examination, and social activities. The results revealed that the L1/L2, L2/L3, and L3/L4% disc heights had a significant relation to knee pain. And among the above discs, there was especially a strong relation between the L2/L3 disc height standardized by the L3 vertebral height and the medial knee space in this study.

Recently, the concept “knee-spine syndrome” has been advocated [11, 12]. Tsuji, et al. stated that in elderly people, decreasing lumbar lordosis and sacral inclination lead to increasing thigh muscle tension and knee flexion while standing, and this increases lumbar back pain and patellofemoral joint pain [11]. On the other hand, Murata, et al. reported that the symptoms from the lumbar spine may be caused by degenerated change in the knee from the analysis of radiological examination [13]. Moreover, Sarwahi, et al. showed from the study of gait analysis of flat back patients, loss of lumbar lordosis causes anterior displacement of center of gravity, which creates instability and increases the work of gait [14]. For elderly people, the ability to compensate is limited by the development of disc degeneration, decreased muscle strength, and decreased overall joint flexibility, it changed the parameters during walking and standing; the hip and knee flexion increased during stance; hip and knee extensor moments were decreased with vertical ground reaction force showing slower rate of loading; reduced peak values; and flattening of normal loading response. Certain gait changes seen in patients with flat back are similar to those seen in patients with osteoarthritis of the knee joint [14]. In our study, it was figured out that decreasing L1/L2-L3/L4 disc height strongly correlated with knee pain, especially L2/L3 disc height had strong correlation. Focusing on L1/L2-L3/L4 disc height might be helpful to investigate the pathology of knee-spine syndrome. Further study should be performed to clarify the cause of these localizations in lumbar spine associating with knee pain.

Fogel and Esses stated that the symptoms of midlumbar spinal stenosis, radiculopathy or neurogenic claudication, may be similar to the pain of an arthritic hip or knee [15]. Hirabayashi,F, et al. reported among 17 patients, 4 were misdiagnosed elsewhere and received conservative treatment for hip and/or knee joint disease [16, 17]. To think about the results from our study that decreasing L1/2-L3/4 disc height, especially L2/3 disc height, were associate with knee pain significantly, we cannot deny that the patients with the knee-spine syndrome include patients caused the knee pain due to radiculopathy associated disc degeneration much more than we expected.

This study has several limitations. First, because we analysed merely the baseline data for this study, this study was a cross-sectional study. To analyze the cause-result relationship between knee pain and decreasing of lumbar disc height, we should perform further analysis using the data at 6 month, 12 month, 18 month, 24 month. Second, the participants in this study are elderly, so there is possibility that knee pain just coexist with decreasing of lumbar disc height without any associations. We have also collected additional data such as blood testing, X-ray imaging of the knee, and joint contracture of the knee and hip, and data at 6 month, 12 month, 18 month, 24 month in LDP study. Further investigation with these data may be helpful to investigate it.

## Conclusions

We found the T12/L1% disc height had a relation to buttock pain and previous vertebral fractures, and also the L1/L2, L2/L3, and L3/L4% disc height had a relation to knee pain. Among these discs, there is an especially strong relation between the L2/L3% disc height and knee pain. It was suggested that these findings may provide additional basis to the concept that lumbar spinal lesion associates with knee pain clinically. We should continue comprehensive analyses for such important issues as back pain in the elderly.

## Additional file

Additional file 1: Interviewer-administered questionnaire. Participants completed an interviewer-administered questionnaire consisting of eighteen items, which included questions on living environment, past medical history, drug history, and pain area (back pain, buttock pain, thigh pain, knee pain). (DOCX 77 kb)

## Data Availability

The datasets generated during and/or analyzed during the current study are available from the corresponding author on reasonable request.

## Abbreviations

AIC: Akaike Information Criterion
CATDAP2: CATegorical Data Analysis Program 2
GLFS-25: Geriatric Locomotive Function Scale-25
LDP: Locomotive Disability Prevention
