# Supplementary material for: Relationship between X-ray findings of lumbar spondylosis and knee pain
Source: BMC Musculoskelet Disord. 2019 Aug 17;20:379. doi: 10.1186/s12891-019-2755-1 (PMC6698333; doi:10.1186/s12891-019-2755-1)
Supplement: Supplementary file 1 — Interviewer-administered questionnaire. Participants completed an interviewer-administered questionnaire consisting of eighteen items, which included questions on living environment, past medical history, drug history, and pain area (back pain, buttock pain, thigh pain, knee pain). (DOCX 77 kb) [file 12891_2019_2755_MOESM1_ESM.docx]

Additional file 1

**Interviewer-administered questionnaire**

Please fill in by asking patient or his well-known family.

Please check those that apply

1. **Basic information**
2. What is the reason for coming to this facility? (Multiple answers allowed).

_ Having some complaint

_ No complaint

_ I am in a nursing facility for elderly.

1. What is the chief complaint? (Multiple answers allowed).

_Lumbar back pain

_Knee pain

_Numbness on the lower extremities

_Pain of the lower extremity on areas other than the knee

_Abnormal posture

_Abnormal gait

_Trauma

_Others

1. Diagnosis of current chief complaint. (Multiple answers allowed).

_Osteoarthritis of the knee

_Osteoarthritis of the hip

_Rheumatoid arthritis

_Other joint disease

_Lumbar back pain

_Spondylosis

_Myelopathy

_Hip fracture

_Fracture of the lower extremities

_ Fracture of the upper extremities

_ Fracture of the spine

_Osteoporosis

_Others

1. Treatment for current chief complaint. (Multiple answers allowed).

_None

_Physiotherapy

_Prosthesis

_Patient education

_Oral medicine

_Injection or block

_Exercise

1. Comorbidity other than musculoskeletal disease. (Multiple answers allowed).

_Hypertension

_Diabetes mellitus

_Disorder of lipid metabolism

_Bronchial asthma

_Chronic respiratory insufficiency

_Renal dysfunction

_Malignant tumor

_Sequelae aftereffect of stroke

_Neuromuscular disease

_Collagen disease

_Cataracts

_Others

1. Medicine currently prescribed

_Antihypertensive agent

_Hypoglycemic agent (including insulin)

_Medicine for disorder of lipid metabolism

_NSAIDs

_Medicine for osteoporosis

_Steroids

_Hypnotic drug

_Antidepressant drug

_Others

1. Past disease which symptom was stabilized. (Multiple answers allowed).

_Stroke

_Cardiovascular disease

_Respiratory disease

_Malignant tumor

_Gastrointestinal disease

_Renal disease

_Fracture of the upper extremities

_Fracture of the spine

_Fracture of the lower extremities

_Arthritis

_Urologic disease

_Trauma of musculoskeletal organ

_Others

**II. Living environment / Past history**

1. What was your job in which engaged for longest period? (Please choose one).

_Self-owned business (Sales)

_Self-owned business (Productive industry)

_Self-owned business (Service)

_Managerial position of company

_Public officer

_Agriculture and forestry

_Fishing industry

_Transport

_Factory production

_Full-time homemaker

_Unemployed

_Others

1. Which was your age of last education background?

_15-17 years old

_18-19 years old

_20-21 years old

_22-23 years old

_More than 23 years old

1. How many family members do you have living together with you?

_None

_1

_2

_3

_More than 3

1. Who are the family members living together with you? (Multiple answers allowed).

_Parent

_Brothers or sisters

_Spouse

_Children

_Other relatives

_Others

1. What type of residence do you live in?

_One story house

_Multiple storied house

_Apartments house with elevator

_Apartment house without elevator

_Others

1. Do you have certification of elderly person's Long-Time Care insurance in Japan?

_Not applied for

_Not applicable

_Unknown

_Needed Support level 1

_Needed Support level 2

_Needed Long-Term Care level 1

_Needed Long-Term Care level 2

_Needed Long-Term Care level 3

_Needed Long-Term Care level 4

_Needed Long-Term Care level 5

1. Were you certified as a specific elderly people of Japanese health care system?

_No

_Yes

_I don’t know

1. Have you ever suffered fracture?

_No

_Yes

_Upper extremity

_Rib

_Spine

_Pelvis

_Proximal femur

_Other lower extremity

1. Have you ever fallen and been injured?

_I never fallen

_I have fallen, but no injury

_I have dropped and suffered injury other than fracture

_I have fallen and suffered fracture

1. Did your health condition change after falling?

_I have never fallen

_I have fallen, but no change of health condition

_ I have fallen and had change of health condition

_Walking disability

_Lumbar back pain

_Aggravation of lumbar back pain

_Lower extremity pain

_Aggravation of lower extremity pain

_Apprehension for falling

_Restriction of going out

_Others

1. Do you use any walking aid? Choose most frequently used one.

_Don’t use

_Use

_Wheel chair

_Walker

_Crutch

_T-shaped Cane

_Long leg prosthesis

_Short leg prosthesis

_Supporter on the knee

_Supporter or prosthesis for the ankle

_Others

**
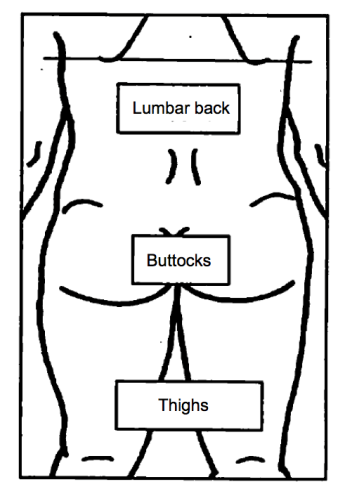
Pain areas**

Referring right figure, check the pain site. (spontaneous pain, pain with motion, tenderness).

|  | Right | Left |
| --- | --- | --- |
| Lumbar back |  |  |
| Buttocks |  |  |
| thighs |  |  |

If the participant has the knee pain, please check. (spontaneous pain, pain with motion, tenderness).

|  | Right | Left |
| --- | --- | --- |
| Knee pain |  |  |
